# A Review of the Sampling, Analysis, and Identification Techniques of Microplastics in the Air: Insights into PM_2.5_ and PM_10_

**DOI:** 10.3390/polym17223045

**Published:** 2025-11-17

**Authors:** Leonela Anahis Solórzano, Dayana Gavilanes, Francisco Cadena, Lourdes Irusta, Alba González, Miguel Aldas

**Affiliations:** 1 Centro de Investigaciones Aplicadas a Polímeros, Departamento de Ciencias de los Alimentos y Biotecnología, Escuela Politécnica Nacional, Ladrón de Guevara E11-253, Quito 170517, Ecuador; leonela.solorzano@epn.edu.ec (L.A.S.); dayana.gavilanes@epn.edu.ec (D.G.); francisco.cadena@epn.edu.ec (F.C.); 2 POLYMAT, Departamento de Polímeros y Materiales Avanzados, Física, Química, Tecnología, Universidad del País Vasco UPV/EHU, Paseo Manuel de Lardizábal, 3, 20018 Donostia-San Sebastián, Spain; lourdes.irusta@ehu.eus (L.I.); alba.gonzalez@ehu.eus (A.G.)

**Keywords:** microplastics, sampling, analysis, identification, air, particulate matter

## Abstract

The massive use of plastics has raised growing environmental concerns, including microplastic (MP) pollution. While most studies have focused on MPs in aquatic environments, research on airborne microplastics has gained increasing attention in recent years. This review discusses the sampling, analytical, and identification techniques used for MPs, with a particular focus on PM_2.5_ and PM_10_ fractions, which have been scarcely addressed in the literature. The main active and passive sampling methods, sample preparation protocols, MP quantification approaches, and chemical characterization techniques applied to airborne plastic particles are compiled. Attention is given to the influence of meteorological conditions on transport and deposition, as well as to the predominant sources of primary and secondary microplastics in both indoor and outdoor environments. The analysis identifies the main research challenges, particularly in the detection of microplastics and in the standardization of protocols. The review highlights the need for standardized methodologies to advance reliable quantification and to better understand the environmental implications of MPs.

## 1. Introduction

Global plastic production reached 400.3 million tons in 2022 [1]. These materials are valued for their durability, cost-effectiveness, and strength. They are widely used in consumer goods and packaging. However, their widespread use has raised concerns about their contribution to global warming and pollution [2]. According to the ISO/TR 21960:2020 [3], microplastics (MPs) are insoluble polymeric materials with sizes ranging from 1 to 1000 μm. However, most scientific studies consider the size range of MPs to extend from 1 μm to 5 mm (5000 μm) [4]. Microplastics can originate as primary particles or can be formed from the use, recycling, or degradation of larger-sized plastics. They come in various shapes, colors, and compositions that change over time, affecting the levels of additives and contaminants [5,6].

While most research has focused on detecting microplastics in aquatic environments, studies on their presence in the atmosphere have been limited due to the difficulty of sampling and analyzing these particles. The first study on microplastics in the air, which examined their abundance, distribution, and composition, was published in 2014 in Lake Hovd, Mongolia [7]. After that, there has been a significant increase in research on this topic since 2019.

**Figure 1:**
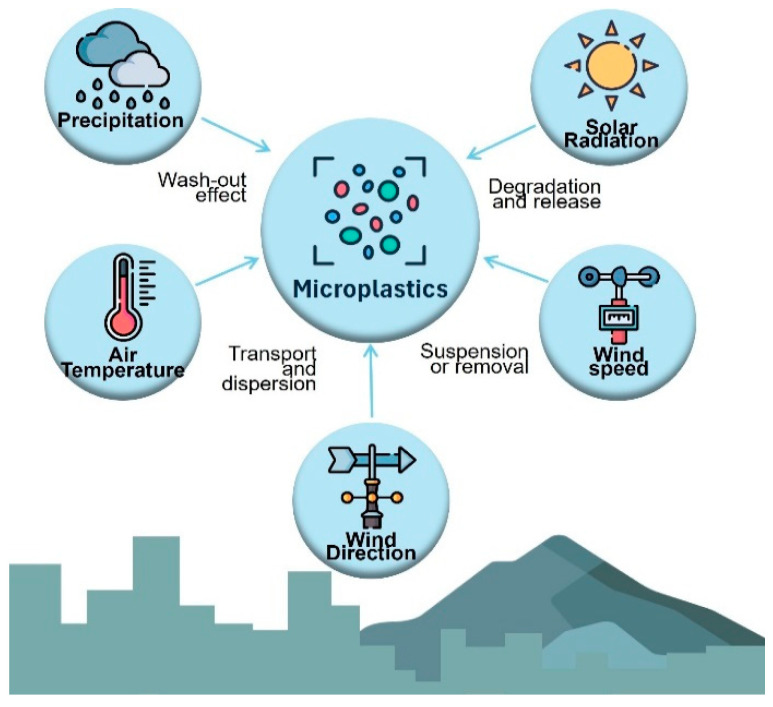
Influence of meteorological conditions on microplastics in the air.

The presence of microplastics in the air is influenced by meteorological factors, such as precipitation, wind direction and speed, topography, and atmospheric pressure, all of which affect dispersion and deposition [8]. Figure 1 shows the influence of meteorological conditions of microplastics in the air. Microplastics tend to accumulate in downwind areas because of their small size and low density [9]. Primary microplastics are produced for commercial products such as cosmetics and textiles. In contrast, secondary microplastics result from the breakdown of larger plastics through physical and chemical processes such as degradation and abrasion [10,11].

This review is novel because it provides a comprehensive overview of airborne microplastic research, focusing on sampling, sample preparation, and identification and quantification techniques. It compiles key variables such as sampling methods, meteorological conditions, and reported results, including microplastic concentration, type, shape, and color. A dedicated section addresses PM_2.5_ and PM_10_ fractions, highlighting their characteristics and implications.

### 1.1. Sources of Emission and Transport of Microplastics in the Atmosphere

**Figure 2:**
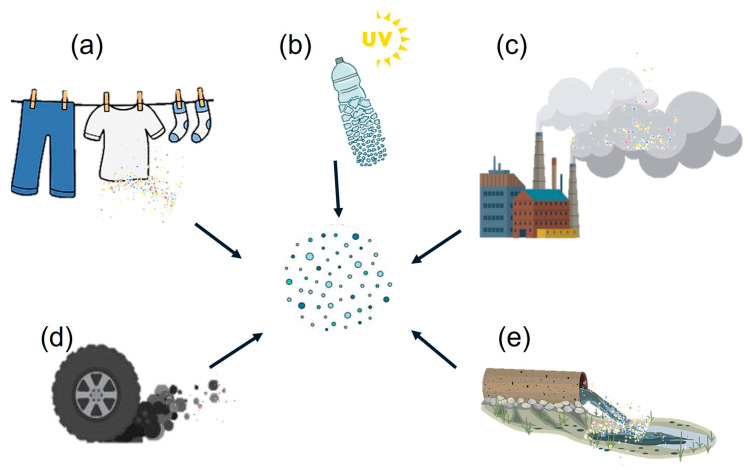
Main sources of microplastic emissions: (**a**) fibers released from clothing, (**b**) fragmentation of plastic containers, (**c**) waste incineration, (**d**) tire wear, and (**e**) industrial emissions.

The primary sources of microplastics in the air are plastic fibers released from clothing and the fragmentation of plastic containers [12]. One cause of microplastic generation is the degradation of larger plastics. Factors such as exposure to heat, humidity, and anaerobic conditions can cause chemical deterioration. This process, combined with wind, can fragment plastics. Additionally, tire wear and activities such as drying synthetic materials release microplastics into the environment [13,14]. Other significant sources are construction materials, waste incineration, and industrial emissions [15]. Figure 2 shows the main sources of microplastic emissions.

Physical and chemical aging play a key role in the atmospheric emission of microplastics. Ultraviolet radiation and oxidants can degrade plastics through multiphase chemical reactions, in which gas-phase or liquid-phase oxidants react with their surface. This process alters their physical properties such as size, shape, morphology, density, and refractive index, and chemical properties such as composition, hygroscopicity, acidity, solubility, and oxidation state [16], leading to an increase in oxygen-containing functional groups that influence their interaction with clouds and radiation. In addition, aging enhances their capacity to adsorb organic contaminants, including per- and polyfluoroalkyl substances and other persistent organic pollutants [17], affecting their stability and environmental behavior.

As mentioned above, there are two types of microplastics: primary and secondary. Primary microplastics are produced on purpose and come from pre-production pellets, compressed air jets, and microbeads in cosmetics, among other similar sources. Secondary microplastics result from the degradation of larger plastics. Their primary sources include fragmented nets and textiles, and effluent treatment from wastewater plants [11].

Because of their small size and low density, microplastics can linger in the atmosphere for a few hours to nearly a week. These characteristics allow them to travel long distances, sometimes even between continents. While the presence of microplastics in the air has been most extensively studied in Europe and Asia, there is a notable lack of data on their distribution in other regions, such as Oceania, Africa, and Latin America. The lack of information highlights the need for more global research [18]. Additionally, the variability in microplastic transport duration and distance depends on local weather conditions, complicating the understanding of their global dispersion.

During transport, microplastics can become electrically charged through friction when contacting other surfaces or particles, which enhances their suspension time. This phenomenon depends on factors such as material type, nature of contact, and relative humidity [19]. According to Chen et al. [20], this behavior can be assessed through the dry deposition velocity (Vd) and gravitational settling velocity (Vg). In the presence of an upward electric field, the Vd/Vg ratio becomes negative, causing particles to rise instead of settling; under a downward electric field, the fall velocity increases, promoting deposition. While particles with greater mass respond more strongly to gravity, lighter particles are susceptible to the electric field due to their high surface-area-to-volume ratio.

### 1.2. Microplastic Air Sampling Process

**Figure 3:**
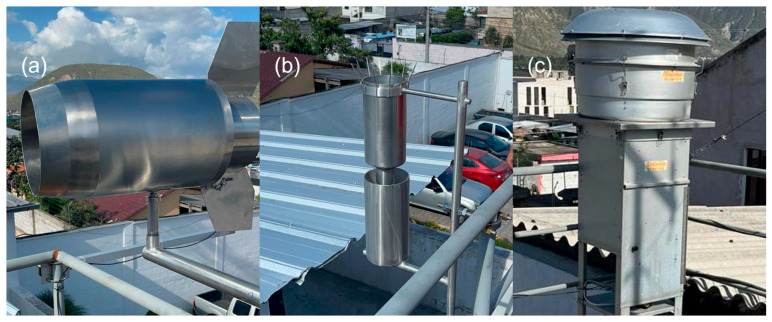
Types of sampling of microplastics: (**a**,**b**) passive sampling, (**c**) active sampling.

There are two primary methods for sampling microplastics in the air: active and passive. Active sampling uses a pumping system to capture air samples, while passive sampling collects deposited particles through rain or dust accumulated on surfaces. Figure 3 shows the types of sampling of microplastics. Passive sampling (Figure 3a) was proposed by Dong et al. [21], while Edo et al. [22] used another type of passive sampling (Figure 3b). The equipment shown in Figure 3a,b was constructed by our team based on the designs and methodology described in the supplementary material from by Dong et al. [21], and Edo et al. [22], respectively.

- Passive Sampling

In passive sampling, microplastics suspended in the air settle due to gravity and weather conditions, allowing them to be collected in atmospheric rainfall through dry and wet deposition. Passive collectors, consisting of stainless-steel funnels and glass bottles, are used for this process. However, aluminum rain gauges can also be used [23]. Researchers like Klein and Fischer [24] have designed alternative collectors using PVC pipes and polyethylene funnels, though this configuration may present cross-contamination risks.

Passive collectors are typically placed outdoors, for example, on building terraces, and samples are collected daily or monthly. It is essential to keep a record of weather conditions to correlate them with the presence of microplastics [25]. After sampling, the funnels and bottles are carefully washed with ultrapure or distilled water. Then, the samples are transferred to containers covered with aluminum foil to prevent contamination.

In addition to rainwater, microplastics can be collected from dust on solid surfaces using a brush and a metal collector. The material is then transferred to an airtight bag for analysis [26]. Indoor sampling usually lasts two to five days [27], while outdoor dust sampling is less frequent [28]. In protected environments, some studies use quartz filters in Petri dishes to capture airborne deposits [26].

J. Zhu et al. [27] placed stainless steel containers measuring 32 cm in diameter and 11 cm in height on a desk 1.2 m above the ground. Edo et al. [22] and Kernchen et al. [29] designed a sampler consisting of a glass bottle collector and an aluminum or stainless-steel frame. They changed the bottles according to the intensity of the rain. In some cases, it is essential to distinguish between dry and wet depositions. For instance, Brahney et al. [30] used equipment with optical sensors in Colorado, USA. These sensors detect rain and activate the corresponding collector, which closes when the precipitation stops.

Passive sampling has several advantages, including ease of collection and low cost. These features make it ideal for long-term studies and hard-to-reach locations. However, its effectiveness can vary due to weather, altitude, and the sampling period. These factors could result in underestimating or overestimating microplastic concentrations [31].

b. Active Sampling

Active sampling uses an operational pumping system with a pump (usually a vacuum pump) and a device or tube with filters. The pump’s air intake can be adjusted according to the study’s specific needs, and the height of the sampler is significant to consider if human health implications are being assessed [15].

This method is more convenient because it adapts to climatic variations, enabling continuous collection over extended periods. It provides a more complete microplastic composition profile than passive methods, which rely on particulate matter deposition processes [32]. The choice of filter type and pore size varies by study. Options include quartz filters, cellulose nitrate filters [33] and GF/A fiberglass filters [15]. Regarding pore size, most studies use 1.6 µm filters [34].

Additionally, some systems use cascade impactors to collect particles of different sizes in separate filters. Separating the particles facilitates the identification of microplastic concentrations by size [29]. An alternative method for active sampling in indoor spaces is to use a vacuum cleaner with samples collected in plastic bags. In Denmark, a thermal respiratory manikin connected to a system that simulated human breathing using pneumatic cylinders was used to measure inhaled microplastics indoors. The manikin had 0.8-μm silver filters and was heated to mimic body temperature. The experiment allowed the detection that air pollution goes from the floor to the mouth or nose. This configuration enabled a more precise evaluation of human exposure to pollutants [35].

Active sampling is a key tool for assessing microplastic pollution and its impact on human health and the environment because it provides more accurate and representative data [32]. However, according to Azari et al. [34], it must have complete data on the sampling flow rate, volume of air sampled, and sampling duration to apply this method. Most studies do not specify these aspects. Additionally, to ensure the reproducibility of this sampling method, a minimum air volume of 70 m^3^ must be collected [32]. While active sampling is efficient and allows for rapid particle collection, it requires a power source, making it more complex and costly due to the need for periodic calibrations to ensure data accuracy [14].

When conducting active sampling, it is crucial to consider the various factors that could affect the representativeness of the data. These include the location, height, sampling period, and weather conditions, all of which must be carefully evaluated. Additionally, adequate wind speed is necessary to facilitate particle capture. To reduce bias and increase the reliability of the results, it is recommended to collect at least three samples per site and randomly select two filters for analysis [36].

On the other hand, the continuous flow sampler for microplastics (FTS-MP) is worth highlighting. This passive sampling device mimics the operation of an active sampler but has a more economical and accessible design. Despite its simplicity, the FTS-MP has been shown to produce results comparable to those of active systems, such as HiVol. Its aerodynamic structure consists of two concentric stainless-steel tubes that automatically orient themselves in the direction of the wind thanks to a set of fins and a rotating bearing, allowing efficient air capture [21].

Innovative methods continue to be developed in the pursuit of more accurate quantification of microplastics (MPs). For instance, Beres et al. [37] achieved real-time quantification using an inline air-flow cytometer, which, combined with machine learning, enabled the identification of individual particles belonging to five polymer types. The system employed holographic imaging and autofluorescence measurements at three excitation wavelengths, achieving a classification accuracy of 90%.

For an accurate assessment of the presence of microplastics in the air, passive and active sampling methods should be used simultaneously [38]. Both can be applied in specific locations and periods; however, they provide different types of information. Passive sampling allows for the estimation of microplastic deposition rates on a surface, while active sampling quantifies the concentration of particles in a defined volume of air. The main disadvantage of passive sampling is that it tends to underestimate the amount of smaller plastic debris, as these particles settle more slowly than larger, heavier ones [39].

c. Sampling height

Depending on the purpose and scope of each investigation, the sampling height varies between studies. In indoor environments, heights between 0.9 and 1.2 m are typically used to simulate the height at which humans breathe [2,10]. Outdoors, various heights have been used, most of which are above 10 m above ground level. However, some studies have taken samples at heights of one and 1.7 m [24,40].

d. Factors related to weather conditions that affect the amount of microplastics that are collected during the sampling process

Weather factors, such as wind speed and direction, temperature, and humidity, directly impact the amount and distribution of microplastics in the air [34]. Most studies have focused on assessing wind speed and direction, finding an inverse relationship between microplastic concentration and temperature and humidity [41]. However, K. Liu et al. [42] reported a positive correlation between barometric pressure and microplastic concentration. Low pressure generates air turbulence, favoring the dispersion of microplastics (MP), while high pressure stabilizes the air, increasing pollution. Depending on wind speed, MP disperse more quickly and can move vertically or horizontally. However, when a thermal inversion occurs, MP become trapped in the lower layers, causing pollution episodes [34].

A study by Chen et al. [43] revealed that microplastic concentrations are higher in poorly ventilated indoor spaces with high carbon dioxide levels. Due to their small size and low density, microplastics can easily be transported by wind and accumulate in large quantities in areas where air currents are directed [44]. Other studies have also analyzed crosswind speed, wind chill, dew point, wet bulb temperature, and heat stress index [34].

The concentration of microplastics varies depending on weather conditions. For instance, Allen et al. [36] observed that polystyrene (PS) concentration increased during periods with fewer storms and rainfall. In contrast, during periods with more precipitation, the concentration of PS decreased, and the polyethylene (PE) concentration increased. In Mexico City, microplastics are more prevalent during the dry season due to wear caused by UV radiation and high temperatures [45]. However, according to Winijkul et al. [46], when comparing the amount of microplastics in atmospheric deposition in Thailand between the wet and dry seasons, a higher concentration was observed during the rainy season. This is because precipitation acts as an atmospheric washing mechanism. Similar behavior was also reported by Prajapati et al. [47], in their study conducted in India.

On the other hand, Liu et al. [48] reported in their study on atmospheric microplastic deposition at Lake Wuliangsuhai that the highest amount of microplastics was found in spring, followed by summer and autumn. Similarly, Zhou et al. [49] observed higher microplastic concentrations in spring and summer, and lower concentrations in autumn. However, in the study by Nafea et al. [50], conducted in the southeastern Yangtze River region, China, the opposite trend was observed: the highest microplastic deposition occurred in autumn, followed by spring and summer. Meanwhile, in the study by Jung et al. [51], it was determined that the highest amount of microplastics occurred in the following order: summer, spring, autumn, and winter. These contrasting results indicate that seasonal trends are site-specific and cannot be generalized to other regions, as they depend on local meteorological conditions, precipitation patterns, and the sources of microplastics present.

### 1.3. Microplastics in Indoor and Outdoor Environments

- Microplastics in Indoor Environments

Microplastics are found in many indoor environments, including homes, offices, schools, hospitals, and transportation systems [2,15,52]. There are no specific regulations or standards for collecting samples in these environments. Passive sampling is common due to its simplicity. However, some studies use active samplers to capture microplastics indoors [14]. In homes, methods such as dust collection using brushes and dustpans, as well as the use of Petri dishes to capture particles, are widely used. For instance, Soltani et al. [2] collected dust in Petri dishes at 32 locations in 22 metropolitan areas in Australia. In another study, Cui et al. [53] measured microplastic pollution in five types of rooms in the homes of 20 families in Yangzhou, China.

Microplastics deposited in enclosed spaces can become airborne during everyday activities. Because dust samples often contain larger particles and fibers, they require pre-filtering before analysis [52]. Indoor dust collection is important because it provides information about indoor pollution and differentiates it from outdoor pollution [31]. To ensure accurate results, tools such as brooms and vacuum cleaners must be used correctly, and the time and place of sampling must be clearly defined. For instance, Zhu et al. [27] collected 242 dust samples using vacuum cleaners, brushes, and dustpans in offices, apartments, hotels, and university classrooms in China. J. Zhang et al. [52] collected 286 samples from 12 countries with brushes to compare the concentration of microplastics.

b. Microplastics in Outdoor Environments

Outdoor microplastic measurements analyze their behavior under various environmental conditions. These measurements are carried out in oceans, mountains, and cities. Active and passive samplers are used. Wet deposition influenced by rain is also measured outdoors.

Outdoors, stainless steel containers or glass bottles are commonly used to collect microplastics. Some studies have compared microplastic concentrations during heavy rainfall versus dry weather. Deionized water is used in the collectors to prevent particles from being resuspended by the wind [54]. In the study by Abbasi [40], the transport of microplastics during a heavy rainfall event was evaluated by collecting samples at 10 min intervals to analyze the dynamics of these particles being carried away under heavy rain conditions. Studies have also investigated the occurrence of microplastics (MPs) in glaciers. Their presence in these environments is primarily attributed to atmospheric deposition, highlighting the transport pathways connecting urbanized and industrial regions with remote glacial ecosystems [55].

Studies by Jia et al. [54] and K. Liu et al. [32] have concluded that active samplers are more effective at capturing microplastics (MP) under any weather conditions, minimizing sample loss and contamination. These samplers enable more accurate analysis of the effects of weather conditions on microplastic deposition and provide continuous collection, offering a more complete profile of outdoor air pollution.

c. A Comparison of Microplastics in Indoor and Outdoor Environments

According to Seo et al. [31], up until 2023, 69.3% of microplastic studies focused on outdoor environments, 19.3% focused on indoor environments, and 11.4% covered both. Simultaneous indoor and outdoor measurement is essential for identifying contamination sources and air exchange rates between the two environments, as they are connected through doors, windows, and ventilation systems.

To effectively compare the two environments, using active sampling equipment with identical operating parameters, including sampler height, filter type, and pumping speed, is crucial. For instance, Choi et al. [56] used the same active sampling equipment to simultaneously collect indoor and outdoor samples in Seoul. Similarly, Dris et al. [57] conducted sampling outdoors for 10–40 h and indoors for 4–7 h on the roof of Paris-EST University, in addition to passive sampling that lasted between four and 15 days.

Studies have shown that the concentration of microplastics indoors can be 6 to 6.5 times higher than outdoors [57,58]. In open spaces, the concentration of microplastics is influenced by factors such as wind direction and speed, temperature, and precipitation. In contrast, the concentration of microplastics in enclosed spaces depends on factors such as the building’s location, number of inhabitants, heating and ventilation systems, and human activities [12].

### 1.4. Sample Preparation and Processing

During sample preparation, it is recommended that you work in a fume hood and wear an apron and cotton or non-synthetic polymer clothing, along with nitrile gloves [59]. Non-plastic laboratory tools, preferably glass, must be used to minimize contamination risk. To ensure complete recovery of microplastic particles, most studies recommend performing at least three rinses of all materials with distilled water [14]. For example, one study rinsed glass materials three times with filtered Milli-Q water, followed by HPLC-grade methanol, before subjecting them to a thermal treatment at 150 °C for three hours [60].

For any analytical technique, it is essential to perform a blank analysis using a clean filter or Petri dish to minimize potential errors in the results [61]. Additionally, the laboratory facilities intended for analysis should be carefully evaluated to identify possible sources of contamination. For instance, Paiva et al. [62] conducted blank controls over three months before initiating the study. Based on this evaluation, several corrective measures were implemented, including filtering distilled water through a 15 μm mesh; covering surfaces and furniture with 100% cotton cloth; disconnecting the air conditioning system; minimizing sample exposure to the environment during processing; and restricting access of personnel not involved in the experimental procedures.

Sample preparation is a critical step in microplastic research because it is necessary to separate these particles from environmental matrices for accurate analysis. As shown in Table 1, different processes can be applied. These matrices may contain minerals, pollen, fungi, and microorganisms [63]. For samples collected by deposition (dry or wet), filtration is typically used to isolate microplastics by size and remove coarse impurities [27]. Stainless steel filters with a pore size of less than 5 µm are recommended, as this material is inert to plastics [64].

Some studies recommend using an ultrasonic bath on filters used for active sampling because organic matter, particles, and microplastics tend to adhere to their surfaces. For instance, Chen et al. [14] recommend a 5 min ultrasonic bath to remove adsorbed material efficiently. Conversely, Akhbarizadeh et al. [71] used 40 min of sonication to quantify microplastics (MP) in PM_2.5_ filters. However, Schrank et al. [72] caution that prolonged sonication can fragment MP due to intense mechanical force, causing fibers to detach from the membrane and making visualization difficult during quantification. This observation is consistent with Corami et al. [73], who applied a sonication time of only 10 s. Despite these limitations, Din et al. [74] emphasize that sonication is essential for releasing and identifying microplastics on the membrane.

Studies aimed at detecting microplastics focus on removing naturally occurring matter through oxidation processes using hydrogen peroxide (H_2_O_2_) or sodium hypochlorite (NaClO) [24]. The most used oxidizing agent is H_2_O_2_ at 30%, due to its easy availability and high oxidative power. Samples are usually treated for 24 h at temperatures between 60 and 70 °C. However, no significant changes in polymers have generally been observed, though some polyamide (PA) modifications have been reported [75]. Additionally, oxidation times range from 12 to 48 h [76,77] or are prolonged to 7–10 days [78,79].

According to Schrank et al. [72], slight alterations in the structure of polystyrene (PS) and polyvinyl chloride (PVC) can be observed, particularly when higher reaction temperatures and longer exposure times are used. Therefore, careful monitoring of the treatment conditions is essential. During the oxidation process, samples with a high organic load may require cooling, as the reaction is exothermic and can cause thermal degradation of microplastics if temperatures exceed 60 °C [80]. As previously mentioned, another reagent for the oxidation process is sodium hypochlorite, which is used at concentrations of 6–14% *v*/*v* to facilitate microplastic extraction.

Fenton’s reagent, consisting of ferrous sulfate (providing Fe^2+^) and hydrogen peroxide, has been shown to digest organic matter more effectively than H_2_O_2_ alone [33]. It is particularly effective at breaking down resistant organic compounds, such as chlorinated aromatics. It is prepared by dissolving ferrous sulfate pentahydrate in an acidic medium [81]. The mixture turns from amber to pale yellow when the reaction with the Fenton reagent is complete [29].

According to Hurley et al. [75], Fenton’s reagent is less aggressive than hydrogen peroxide because it does not cause surface degradation in polymers or significant color changes. It is recommended that the temperature does not exceed 40–50 °C when using this reagent, as maintaining milder conditions minimizes the risk of polymer damage [80]. However, it can cause minor damage to biodegradable polymers, such as polylactic acid (PLA) [82]. Adding ascorbic acid can increase the effectiveness of the reaction and improve the decomposition of organic matter. An orange precipitate may also form during the process, and it can be removed by adding citric acid or by using density separation [72].

On the other hand, alkaline digestion is another method to break down biogenic matter, including proteins, lipids, and carbohydrates, into a low-molecular-weight aqueous solution. Sodium hydroxide (NaOH) and potassium hydroxide (KOH) are commonly used; the latter is the most effective and powerful maceration agent [72]. Additionally, some studies combine KOH with hydrochloric acid and pentanol. In this combination, KOH facilitates the decomposition of the organic matrix, while pentanol acts as a solvent, optimizing digestion [70,83]. However, the main limitation of this type of pretreatment is the possible degradation of specific microplastics. Hurley et al. [75] found that exposure to a 10 M NaOH solution at 60 °C for 24 h significantly decreased the size and weight of PC and PET microplastics. The chemical structure of PC and PET makes them susceptible to saponification; PET also undergoes surface erosion.

Using more diluted alkaline solutions, such as 1 M NaOH or 10% KOH, reduces degradation; however, it remains significant, resulting in a mass loss of around 16% [72]. In the alkaline digestion process, it is crucial to consider the exposure time and temperature. For instance, Hurley et al. [75] observed slight surface alterations in PA, PET, PC, PVC, and PMMA microplastics when comparing a 5 h exposure to concentrated KOH (20 M) at 20 °C with a 20 min exposure at 80 °C.

In addition to alkaline digestion, there are acid digestion methods. According to Schrank et al. [72], using nitric acid (HNO_3_) at a concentration of 15.7 M at 80 °C for two hours is highly effective at destroying biogenic matter but extremely destructive to certain microplastics, such as PA, PET, and PUR. Conversely, the combination of HNO_3_ and HClO_4_ has been shown to significantly degrade and alter the surfaces of polymers such as PA, PUR, ABS, PMMA, and PVC [84]. Other acids include peroxomonosulfuric acid (H_2_SO_5_) and hydrochloric acid (HCl). When used at 20.5 °C for 48 h, H_2_SO_5_ causes significant degradation in PA and PUR [72]. In contrast, HCl is less efficient at removing organic matter. For instance, 37% HCl at 25 °C for 96 h degraded PA, altered the surface of PVC, and caused PET to agglomerate [85]. Although strong acids are effective at digesting biogenic matter, they can also severely damage microplastics, skewing the results of analyses.

Another alternative for removing organic matter is through enzymatic purification. Enzymatic methods using protease K, cellulase, and chitinase are highly effective at removing biogenic matter without causing damage to microplastics. However, these methods require prolonged incubation and careful handling to avoid particle loss [72]. At an intermediate stage of the process, Fenton’s reagent can be incorporated to mitigate damage to polymers, considering the possible degradation of polylactic acid [80].

**Figure 4:**
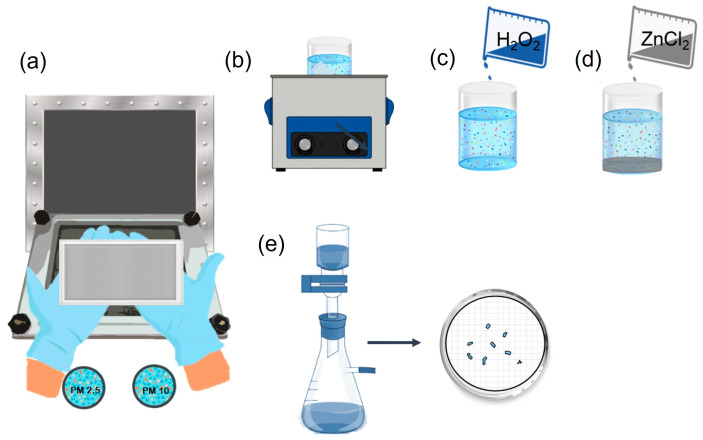
Sample treatment: (**a**) collection, (**b**) sonication, (**c**) oxidation, (**d**) density separation, and (**e**) vacuum filtration.

Before removing organic matter, it may be necessary to separate microplastics from metals and silicates. Saline solutions, such as zinc chloride (ZnCl_2_), are effective for this purpose due to their high density (between 1.6 and 1.8 g/cm^3^), which facilitates the separation of denser particles [31]. It is recommended that 15 mL of concentrated zinc chloride solution be placed in 20 mL glass vials to accomplish this. The vials are then sealed with aluminum foil and kept at room temperature for 48 h [72]. However, zinc chloride has a negative environmental impact. Solutions like sodium chloride or sodium bromide have a lower ecological impact; however, their lower density may limit their ability to separate higher-density microplastics. KI and NaI solutions have also been used; however, their higher cost makes them less accessible [86,87]. According to Zobkov & Esiukova [88], some of these salts are corrosive and acidic, which could affect specific polymers. However, Schrank et al. [72] found that using zinc chloride at a density of 1.8 g/cm^3^ for 48 h at room temperature did not result in visible degradation of the analyzed polymers. Nevertheless, it is essential to minimize the exposure time of the samples to these solutions to prevent potential damage to the microplastics. Figure 4 shows a scheme of sample preparation and processing.

As previously mentioned, using strong reagents and high temperatures (above 50 °C) can accelerate the degradation of microplastics (MPs). Consequently, novel and less aggressive techniques, such as oil-based extraction (oleoextraction), have been developed. This method allows the separation of microplastics across a wider density range, including polymers with densities around 2 g/cm^3^ and other plastic additives, natural fibers, and non-plastic materials [89]. Table 2 presents the advantages and disadvantages of the different sample preparation methods.

During oleoextraction, the sample is mixed with sunflower seed oil and hydrogen peroxide (H_2_O_2_) to form an emulsion, which is then allowed to settle to facilitate phase separation; plastic particles concentrate in the oil phase. Subsequently, this oil phase is recovered by adding hexane and ethanol, followed by filtration and purification with alternating washes of ethanol, methanol, and hexane to remove residues and interferents [73]. This approach is preferable to flotation with saline solutions, as it prevents the accumulation of salts that can hinder polymer identification in subsequent analysis stages [73].

The solution resulting from the organic matter removal and density separation processes is stirred by centrifugation, after which it is vacuum filtered. This process is repeated thrice to ensure adequate separation [40]. The sample is then dried at room temperature. Glass fiber or Teflon (PTFE) membranes with a pore size of 0.45 µm are commonly used for vacuum filtration [71,87]. Sometimes, the filters are immersed in distilled water or ethanol before ultrasound treatment to remove suspended material from the microplastics’ surface [23,33,65].

### 1.5. Microplastic Identification Techniques

Microplastics are identified through morphological and chemical analyses. Morphological analysis evaluates abundance, size, shape, and color characteristics. Chemical analysis, on the other hand, identifies the composition of polymers. The most used techniques are Fourier-transform infrared (FT-IR) spectroscopy and Raman microscopy for chemical analysis, and stereomicroscopy and scanning electron microscopy (SEM) for morphological analysis.

- Visual Analysis

Visual analysis involves identifying microplastics by observing their size, shape, and color under optical microscopes such as fluorescence, binocular microscopes, or stereomicroscopes. This technique is helpful for quickly quantifying large quantities of microplastics. However, this method has significant limitations, such as difficulty identifying white, transparent, dark, or translucent microplastics. Visual analysis also becomes particularly complex for particles smaller than 50 µm [14,90]. While stereomicroscopes make distinguishing between natural and artificial fibers difficult, they are the most direct method for manually counting microplastics [91]. According to Bhat et al. [60], the filter should be divided into eight sections for stereomicroscope analysis, and counting should be performed three times to minimize human bias.

However, high-resolution visual inspection is possible using high-magnification optical microscopy methods and optimized lighting techniques. For example, in a study by Gaston et al. [58], microplastics as small as 20 μm were detected using an Olympus SZ61 stereomicroscope. According to Yang et al. [91], the identification of microplastics depends on the observer’s skill. The same author points out that when identifying particles smaller than 50 μm, the margin of error is approximately 63%, and the number of false positives increases with the decrease in microplastic size.

An extraction protocol should be established before microscopic analysis to eliminate false positives. According to Löder et al. [92], particles larger than 500 µm are separated into fractions for identification purposes, whereas particles smaller than 500 µm require additional extraction. The following guidelines are suggested for identifying microplastics using microscopy techniques: differentiating between brightly colored and unnatural particles, geometric shapes, shiny surfaces, and smooth fibers of a specific thickness [93]. Hot needle physical tests are also recommended to confirm the presence of microplastics; however, this method only applies to particles larger than 100 µm [94]. The hot needle test involves applying a hot metal needle to suspicious particles. If they melt or deform, they are presumed to be plastic [95].

After identifying the microplastic particles, their size, shape, and color are analyzed. The types of microplastics vary according to these parameters, as well as the detection limits of the equipment and the filters used to extract the particles. The main benefit of using microscopy is its low cost; laboratories already have this equipment and usually only need to add cameras. Additionally, software such as Histolab or ImageJ (v1.54) is used for online analysis [94].

Some studies use the Nile Red (NR) dye to improve microplastics (MP) identification, particularly those smaller than 100 µm that are difficult to see under a microscope. NR is a lipophilic dye that dissolves in organic solvents, such as acetone, n-hexane, and chloroform, and selectively stain microplastics. NR concentrations range from 10 µg/mL to 1 mg/mL [15,23,24]. NR is handy for visualizing transparent microplastic fibers [2]. The filter is stained with NR, visualized using fluorescence techniques, and analyzed by manual or automated counting [94]. However, according to Yoo et al. [96], relying solely on staining methods can lead to overestimating microplastic (MP) counts. For instance, only 27% of the stained particles were confirmed as plastics, while 73% originated from tire or road wear.

Necessary precautions during stereomicroscope analysis include cleaning the surface where the sample will be placed using 70% alcohol. Additionally, three Petri dishes filled with distilled water should be positioned around the stereomicroscope to monitor potential environmental microplastic contamination. These Petri dishes must be pre-washed three times with distilled water [62].

b. Scanning Electron Microscopy (SEM)

Scanning electron microscopy (SEM) is a widely used technique for identifying microplastics. SEM provides high-resolution surface images by interacting with a sample using an electron beam. This technique allows the surface characteristics of microplastics, such as color, shape, and size, to be compared. The detection limit of SEM is 20 nm [34]. However, SEM requires time-consuming sample preparation, such as applying a conductive coating. Therefore, it is less suitable for analyzing large quantities of microplastics [97].

Studies can examine surface properties, morphology, and the effects of artificial aging or biodegradation in microplastic identification. An energy-dispersive X-ray spectrometer can measure the radiation emitted when the beam interacts with the surface. Additionally, organic contamination can interfere with the analysis by depositing amorphous carbon on the sample [98].

c. Fourier Transform Infrared Spectroscopy (FTIR)

Fourier transform infrared spectroscopy (FTIR) is the most widely used analytical technique for determining the composition of microplastics. FTIR provides a unique spectrum for each analyzed sample, enabling identification of the microplastic type by comparing the obtained spectra with those in databases. This non-destructive technique can analyze small particles with greater precision than visual analysis. In environmental studies, attenuated total reflectance (ATR-FTIR) is commonly used for identifying microplastics [99,100]. For microplastics in the air, FTIR microspectroscopy (μ-FTIR), which combines an FTIR spectrometer with a microscope, is frequently used to detect particles up to 20 µm. This technique allows for the individual analysis of small particles by identifying their chemical composition [14]. For μ-FTIR analysis, particles are sorted onto reflective slides to measure each microplastic individually [94].

The study’s objective generally determines the type of FTIR analysis used, but in most cases, microplastics are first separated by size. They are typically classified as either smaller than or larger than 500 µm. FTIR microscopy coupled with ATR also enables measurement of particles on slides, compression cells, windows, and filters; however, it has long measurement times and contamination risks. Transmission FTIR microscopy may not be suitable for large particles because they can block the light beam completely. In contrast, reflection mode requires particles to have good reflective properties and is less effective for small or dark particles. Particles smaller than 500 µm are concentrated on aluminum oxide filters, which are widely used in recent studies along with metal-coated polycarbonate (PC) filters and silicon membranes for FTIR [90].

LDIR-FTIR spectroscopy is an advanced, FTIR-based technique designed to detect and characterize microplastics in environmental settings. Unlike conventional FTIR, LDIR-FTIR offers greater sensitivity and specificity in identifying microplastics in complex samples, such as air. However, it cannot differentiate between polymers such as PET and PU [67].

d. Raman Spectroscopy

Raman spectroscopy is another popular microplastic detection technique [101]. Like FTIR, Raman spectroscopy can analyze small amounts of microplastics. Raman microspectroscopy (μ-Raman) can detect particles as small as 1 µm [25]. However, this technique has limitations. For example, it requires the removal of all organic matter or dyes to avoid high background fluorescence and possible [14,102]. Despite these challenges, Raman spectroscopy is highly sensitive to nonpolar functional groups, reducing the effects of interference from moisture [100]. Raman spectroscopy can complement FTIR [103].

**Figure 5:**
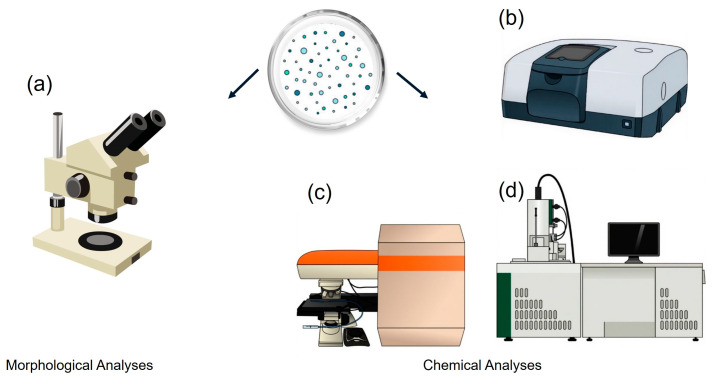
Microplastic identification techniques: (**a**) Stereomicroscopy, (**b**) FTIR spectroscopy, (**c**) Raman microspectroscopy and, (**d**) SEM.

According to Primpke et al. [94], three different approaches can be used to analyze microplastics with Raman spectroscopy. The first approach involves identifying microplastics as individual particles. In this approach, particles larger than 300 µm must adhere to transparent tape or aluminum foil to avoid background fluorescence. Particles smaller than 300 µm must be measured directly on the filter [104]. Figure 5 shows some microplastic identification techniques.

The second approach is based on the automated identification of particles using the Particle Finding algorithm. When microplastics are smaller than 20 µm, the particles must be isolated using membrane filtration. The membranes must be inert, homogeneous, and free of fluorescence interference within the polymers’ absorption range. Therefore, metal-coated polycarbonate (PC) or silicon membranes are commonly used [105]. A promising approach is the automated identification of particles using image analysis software, which improves the detection and measurement of particles. The information recovered includes the number, size, and shape of MP. First, all particles in a selected filter area are detected using image processing criteria (black/white contrast). Then, the detected particles are automatically measured with RM. A third approach involves point-by-point mapping with “Imaging Mode,” which acquires spectra in a defined grid with signal enhancement and noise removal using EM-CCD detectors. This approach is faster but limited to particles of 12 µm [70,103].

e. Other Identification Techniques

Microplastic identification techniques include thermal degradation methods, such as pyrolysis gas chromatography/mass spectrometry (Pyr-GC/MS) and thermogravimetric analysis/mass spectrometry (TGA-MS). However, these techniques are destructive, so they cannot determine the shape and size of microplastics [106]. Pyr-GC/MS breaks down the sample into smaller, more volatile compounds separated by gas chromatography and identified by mass spectrometry. Using a low-temperature thermal desorption process, this method helps detect low concentrations and analyze additives, plasticizers, pigments, and flame retardants in plastic [107]. The thermal decomposition and pyrolysis products vary depending on the polymer type, ranging from the most complex (e.g., PE, PP, and PET) to the simplest (e.g., PMMA), generating characteristic patterns based on the applied temperature. Pyrogram and spectrum databases allow the reliable identification of over 165 common polymers [108]. However, not all polymers can be identified due to variability in decomposition spectra and thermal stability [90].

Furthermore, Pyr-GC/MS is slower than techniques such as FTIR and Raman, taking around 30 min per sample because it analyzes one particle at a time. To identify microplastics faster, EGA-MS with short silica capillary tubes is recommended. However, although only a small sample is needed, the particles must be larger than 100 µm to fit in the pyrolysis tube [108].

### 1.6. Types of Microplastics Found

As shown in Table 3, no single type of plastic predominates in all studies. The prevalence of the types of plastics found depends on the city and country in which sampling was conducted. According to Noorimotlagh et al. [12], PET, PP, PE, and PES exhibited the highest detection rates in studies conducted in both indoor and outdoor environments. The high prevalence of PET and PE is primarily due to high consumption of packaging products [86]. For instance, PET fibers were identified in urban areas, primarily in products such as plastic bottles, containers, and packaging films. Due to UV radiation and other degradation mechanisms, these products fragment and release microplastics [109]. Meanwhile, PE fragments were mainly found in Shihezi (China) [86].

On the other hand, polypropylene (PP) and polyethylene terephthalate (PET) are predominant in microplastic concentrations due to their presence in textiles and clothing [54]. PP stands out for its resistance to UV radiation and moisture, facilitating its environmental dispersion and accumulation. PS is generally found in street dust and originates from industrial emissions and the degradation of packaging. It is durable and lightweight, enabling it to travel long distances [31].

In agricultural areas of Cartagena, PS was the most detected microplastic due to the use of inorganic and organic materials and wastewater treatment plant fertilizer residues [106].

### 1.7. Identification of Fibers, Fragments, Spheres, and Films—Colors of Microplastics in the Air

Microplastics are tiny particles that come in various shapes and sizes. They can be fibers, fragments, spheres, or films. Fibers are elongated and thin, resembling microscopic threads or hairs. Fragments are irregular pieces of plastic that may have sharp or rounded edges. Microplastic films appear as thin, transparent sheets that are often flexible and small. Finally, spheres are small, solid particles ranging from a few micrometers to millimeters. According to Noorimotlagh et al. [12], microplastics in indoor environments generally range from 0.4 µm to 8 mm, while microplastics in outdoor environments generally range from 2 µm to 10 mm.

Microplastic fibers are the most abundant type of microplastic in indoor and outdoor environments, followed by fragments, films, and spheres. Primary microplastics maintain their morphology regardless of environmental conditions [12]. The overuse and washing of synthetic textiles generate fibers in indoor spaces. Fragmented microplastics originate from the decomposition of plastic products due to exposure to UV radiation and wear and tear [110]. Fragmented microplastics predominate in street dust, likely due to the fragmentation of vehicle tires [86]. High levels of fragmented microplastics are observed in areas close to marine or mountainous environments, probably due to transport from urban areas [36,111].

Microplastics come in various colors, including white, transparent, black, red, pink, green, yellow, orange, blue, brown, purple, and gray. In indoor environments, the most predominant colors are transparent and white. In outdoor environments, black and blue predominate [12].

### 1.8. Microplastics Concentration

**Figure 6:**
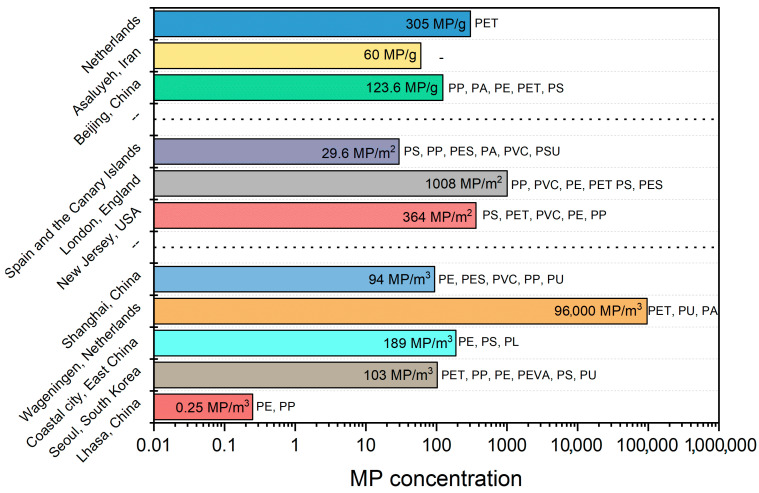
Concentrations and most frequent types of microplastics in different cities.

The concentration of microplastics in indoor and outdoor environments varies depending on the study site and sampling method. Noorimotlagh et al. [12] found that microplastic concentrations in studies can be expressed as follows: (a) MPs/m^3^, (b) MPs/m^2^, (c) MPs/g of particulate matter (PM), and (d) mg/g of PM. Figure 6 presents the concentrations and the most frequent types of microplastics in different cities. Additionally, microplastic concentration is directly related to human activity at each site. For instance, a study in China examined apartments, offices, classrooms, hospitals, and transit stations. The study determined that the abundance of microplastics, expressed in MPs/m^3^, was highest in apartments, followed by offices, transit stations, classrooms, and hospitals [15].

As mentioned above, microplastic concentrations are usually higher indoors. The high concentration may be due to the accumulation of fibers and particles in poorly ventilated, closed environments and constant sources such as textiles and household materials. Outdoors, there is a higher risk of fragmentation due to wear and tear and exposure to factors such as movement and UV radiation. However, due to wind and other atmospheric mechanisms, microplastics disperse more easily outdoors [31]. However, the concentration of microplastics is higher in urban areas than in rural areas due to higher population density and industrial activity [43].

### 1.9. Microplastics in the Air and Their Effect on Health

Given their ubiquity and persistence in the environment, airborne microplastics are a growing environmental and health concern. Inhaling these particles is a significant exposure route for humans, especially in urban and industrial areas where concentrations are higher. Studies have shown that microplastics can penetrate the airways, causing inflammation and oxidative stress. This ease of penetration could increase the risk of chronic respiratory diseases such as asthma and pulmonary fibrosis [112,113].

When reporting on the presence of microplastics, it is essential to consider the particles’ length and diameter. Diameter is critical in determining whether particles can be inhaled, while length influences their ability to persist in the body and their potential toxicity [12]. For instance, Jabbal et al. [114] observed that particles smaller than five μm can enter the lungs. The deposition of microplastics in the respiratory system, in either the upper or lower airways, is influenced by the aerodynamic equivalent diameter (AED), which depends on the particles’ density and physical diameter. Lower-density polymers with a small diameter, such as low-density polyethylene (LDPE), have a higher potential to be respirable and reach the lower airways [113].

### 1.10. Determination of Microplastics in PM_2.5_ and PM_10_ Fractions

Due to their potential impact on human health and the environment, the study of microplastics (PM) in the PM_2.5_ and PM_10_ particulate matter fractions is an area of interest. PM_2.5_ corresponds to particles with diameters less than 2.5 μm, and PM_10_ corresponds to particles smaller than 10 μm in diameter. Smaller particles can easily be inhaled, increasing the risk of adverse respiratory effects. However, few studies have addressed identifying and quantifying microplastics in these fractions. For instance, microplastic concentrations have been detected in PM_10_ and PM_2.5_ in cities such as São Paulo and Mexico City. In Mexico City, polymers such as cellophane, polyethylene (PE), and polyethylene terephthalate (PET) were identified [45]. In São Paulo, polyester fibers (PL), PE, and PET particles predominate [41]. The friction and abrasion of tires and textiles are significant sources of airborne microplastic contamination in these fractions. In a study conducted in Austria, microplastics were measured in PM_10_ and PM_1_ fractions, with PET, PE, and PP identified as the most abundant polymers. The highest concentrations were recorded during summer and autumn, and the microplastic concentration in PM_10_ was found to be twice that in PM_1_ [115].

In the study by Kernchen et al. [29], microplastics in the PM_2.5_, PM_10_, and total atmospheric deposition fractions were identified. Raman spectroscopy was practical in analyzing the PM_2.5_ and PM_10_ fractions because it can detect very small particle sizes. This technique allowed researchers to identify potentially respirable microplastics with diameters smaller than 10 μm. Similarly, Akhbarizadeh et al. [71] demonstrated that the micro-Raman technique can identify microplastics with diameters smaller than 2.5 μm. The study also noted that visually identifying microplastics in this size range is complex and that using a fluorescence microscope with up to 400× magnification facilitated identification. Furthermore, plastic particles smaller than one μm are not quantified because this range is outside the lower limit of detection of the object of study.

For the detection of microplastics (MPs) in PM_10_ fractions, it is recommended to combine identification techniques such as fluorescence microscopy, Raman microspectrometry, and scanning electron microscopy to avoid overestimation. In the study by Yoo et al. [96], only 0.008% of the particles collected in PM_10_ were confirmed as microplastics. Wu et al. [116] also recommend using thermal desorption coupled with gas chromatography-mass spectrometry (TD/GC-MS) for quantifying microplastics in PM_2.5_, highlighting polyvinyl chloride (PVC) as the most predominant polymer. Similarly, Costa-Gómez et al. [117] applied thermogravimetric analysis coupled with mass spectrometry to quantify polystyrene microplastics in PM_10_ and PM_2.5_, reporting concentrations of 2.09 and 1.81 ng·m^−3^, respectively.

An innovative study in Jiangwan (Shanghai) collected PM_2.5_ samples using a versatile aerosol concentration and enrichment system (VACES). It concentrates suspended particles up to ten times without altering their physical or chemical composition via a preconcentration process based on inertia and controlled humidified flow. The samples were analyzed using TD/Py-GC-MS in dual-shot mode to identify phthalate esters (PAEs) and fine plastic particles [118].

These fractions are generally collected using active samplers that comply with U.S. EPA reference methods for PM_2.5_ and PM_10_, with a sampling flow rate of 16.67 L/min [119]. To ensure accurate size distribution measurement, particles must adhere to the collection surface after impact. The size separation process is influenced by the particles’ aerodynamic behavior, which is determined by their size, shape, and density [29].

The presence of microplastics in the PM_2.5_ and PM_10_ fractions is related to the aerodynamic diameter of the sampler inlet. In a study by Abbasi et al. that used PM_2.5_ samplers, the number of recovered particles was relatively low. It is important to note that fibers, the predominant form of suspended microplastics, can be much longer than wide, affecting their capture efficiency. Therefore, their orientation in the sampler inlet plays a key role in their collection. After evaluating different inlet sizes, the study concluded that PM_10_ samplers provided more representative samples.

Yao et al. [25] analyzed PM_2.5_ and PM_10_ samples using advanced techniques such as scanning electron microscopy (SEM) and X-ray energy-dispersive spectroscopy (EDS). This analysis revealed various elements present in the collected particles. The PM_2.5_ samples mainly contained the elements C, O, Si, P, and Ca. In contrast, the PM_10_ samples contained Na, Al, Mg, Cl, and S. These common environmental elements can adhere to the surfaces of suspended microplastics. These trace elements could influence the degradation of microplastics and nanoplastics in the air. Common elements that bind to microplastics include Ca, Al, Na, Cl, P, S, O, and C. This information is relevant to understanding how these contaminants interact with their environment and other contaminants in the air. Furthermore, the contribution of tire wear to particulate matter has been estimated to range from 3 to 7% by mass for PM_2.5_ and up to 11% for PM_10_ [120,121].

The presence of microplastics (MPs) in PM_2.5_ fractions has also been investigated, along with their coexistence with ultraviolet stabilizers (UVAs), another emerging contaminant in this fraction. Both MPs and UVAs were found to reach their highest concentrations during winter, likely due to air stagnation. Significant correlations were observed between MPs and PM_2.5_, UVAs, and carbon monoxide (CO), with coefficients of 0.69, 0.58, and 0.58, respectively. These findings suggest that the combustion of plastic waste is the main emission source [122].

Additionally, images obtained by scanning electron microscopy (SEM) and focal plane array microscopy showed that, although relatively small, PM_2.5_ and PM_10_ particles have a texture similar to particles found in total deposition samples. The similarity in texture suggests that although airborne particles are tiny, their structure may be more closely related to larger deposited particles, which could influence their persistence in the environment and their ability to be inhaled [25].

### 1.11. Recommendations and Research Gaps

Based on the information gathered in this literature review, a series of recommendations are established for the collection, preparation, identification, and quantification of airborne microplastic samples. Additionally, the existing research gaps were identified.

- Sampling Location
  Recommendations:
    - Select the sampling location according to the study objective: urban/rural setting, proximity to direct sources of microplastics (MPs), sampling height, and indoor or outdoor environments.
    - Record environmental characteristics: temperature, humidity, atmospheric pressure, wind speed, and direction.
  Gaps: limited information from rural environments; scarce data from Oceania, Africa, and Latin America; and contradictory results regarding seasonal and meteorological variability.
- Sample collection
  Recommendations:
    - Active sampling: use an adjustable air pumping system, recording air volume and sampling time to ensure reproducibility.
    - Passive sampling: collect deposition from rain or dust; this method is ideal for long-term studies and remote locations.
    - It is recommended to use both methods simultaneously.
  Gaps: lack of standardization in filters, pore sizes, and air volumes; although fiberglass filters < 1.6 µm are recommended, there is no universal consensus.
- Sample Preparation
  Recommendations:
    - Use cotton clothing and nitrile gloves.
    - Employ only glass or stainless-steel materials.
    - Perform blank controls to detect cross-contamination.
    - Organic matter treatment: use H_2_O_2_ and Fenton’s reagent with controlled temperature and duration to prevent MP degradation.
    - Alkaline digestion: use ZnCl_2_, carefully controlling concentration, time, and temperature to protect sensitive microplastics (PA, PET, PC, PLA).
    - Sonication: apply short durations to remove particles without fragmenting MPs.
    - Filtration: use fiberglass or PTFE filters with 0.45 µm pore size, followed by air drying.
  Gaps: alternative, less aggressive, and less polluting methods are still underdeveloped; limited information exists on the validation of density separation (oleoextraction) for different polymer types.
- Recommendations for Identification and Quantification of Microplastics
  - Visual Analysis: Use stereomicroscopy (not suitable for particles smaller than 50 µm) and SEM to identify particle size, shape, color, and surface morphology.
  - Spectroscopic Analysis: For chemical identification, use µFTIR and µRaman spectroscopy—the latter for particles smaller than 1 µm.
  - Thermal Analysis: Apply Pyr-GC/MS and TGA-MS to identify plastic additives and polymer composition.

## 2. Conclusions

Research on airborne microplastics (MPs) has advanced significantly; however, challenges remain regarding the comparability of results. This review examined and discussed the techniques employed for sampling, emission and transport sources, sample preparation, quantification, and chemical characterization. It also explored a less-addressed aspect in the literature: their relationship with fine particulate matter (PM_2.5_ and PM_10_).

The discussion highlights that active and passive sampling methods present advantages and limitations depending on sampling duration, MP retention efficiency, and meteorological conditions. Moreover, no standardized protocol exists for the critical step of sample preparation, as chemical digestion and flotation, while effective in removing organic and inorganic interferents, may also degrade certain polymers. The lack of a standardized protocol has motivated the development of novel approaches such as enzymatic digestion and oil-based extraction. Necessary precautions during visual analysis (stereomicroscopy) and the limitations of analytical techniques (µ-Raman, FTIR, pyrolysis-GC-MS) were also identified. Although these techniques offer varying detection limits, they are mainly limited in detecting particles smaller than 1 µm.

The review further identified a lack of standardization in definitions, units, and classification criteria and a scarcity of studies conducted in tropical, rural, or coastal regions. Given that PM_2.5_ and PM_10_ fractions can penetrate the respiratory system, it is recommended that MP monitoring be incorporated into air quality programs.

## Figures and Tables

**Table 1:** Information on methods used in airborne microplastic determination studies.

City	Sampling Environment	Filters	Type of Sampling	Sample Preparation	Identification Methods	Ref.
Lhasa, China	Outdoor	Fiberglass (1 μm) and Polycarbonate filters (1 μm)	Passive	30% H_2_O_2_	u-FTIR, Microscopy	[21]
Beijing, China	Outdoor	-	Passive	30% H_2_O_2_ ZnCl_2_ (1.7–1.8 g/cm^3^) Ethanol and ultrasound washes	SEM, LDIR	[65]
New Jersey, USA	Outdoor and indoor	Quartz filters (2.2 μm)	Active and passive	30% H_2_O_2_	Optical microscopy and stereoscopy Raman	[25]
Asaluyeh, Iran	Outdoor	PTFE filters (2 µm)	Active and Passive	35% H_2_O_2_ NaI (1.6 g/cm^3)^	Binocular microscopy SEM	[64]
London, England	Outdoor	-	Passive	Ultrasonic treatment with HPLC-grade methanol NR addition	Fluorescence microscopy, FTIR	[23]
Seoul, South Korea	Outdoor	Cellulose nitrate filters	Active	Fenton reagent Density separation LMT	uFT-IR microscopy	[33]
Hamburg, Germany	Outdoor	-	Passive	6–14% de NaClO	Fluorescence microscopy Raman	[24]
Sakarya, Turkey	Outdoor	Stainless-steel filters (50 μm and 500 μm)	Active	35% H_2_O_2_ ZnCl_2_ (1.5–1.7 g/cm^3^)	Optical microscopy u-FTIR	[66]
Coastal City, East China	Outdoor and indoor	Whatman glass microfiber filters (0.7 μm)	Active	30% H_2_O_2_ NR addition	u-FTIR	[15]
Wageningen, Amerongen, Zetten and Utrecht. Netherlands	Indoor	-	Passive	96% ethanol treatment	LDIR	[67]
Spain and the Canary Islands	Outdoor	-	Passive	Digestion with 33% H_2_O_2_	Stereomicroscopy u-FTIR	[22]
Netherlands	Indoor	PTFE filters (0.2 µm)	Passive	Ethylene glycol	LC-UV (LOD ¼ 62 mg/L) LC-ESI-MS	[68]
Shanghai, China	Outdoor and indoor	Glass fiber filter GF/A (1.6 μm)	Active	KOH and pentanol	u-FTIR ESIeMS/MS	[69]
Shanghai, China	Outdoor and indoor	Alumina filter GE (0.22 μm)	Active	Dilute hydrochloric acid, pH = 3.	Raman	[70]

**Table 2:** Advantages and disadvantages of microplastic sample preparation methods.

Method	Main Reagents/Conditions	Advantages	Disadvantages	References
Oxidation with H_2_O_2_	30% H_2_O_2_, 60–70 °C, 12–48 h (up to 7–10 days in some cases)	- Easy availability and high oxidative power. - Minimal alteration of most polymers under controlled conditions.	- May cause slight degradation in PA and PVC under prolonged exposure. - Requires careful temperature control due to exothermic reaction.	[24,76,77,78,79,80]
Sodium hypochlorite (NaClO)	6–14% *v*/*v* solution	- Effective for organic matter removal.	- Produces chlorinated by-products	[24]
Fenton’s reagent (Fe^2+^/H_2_O_2_)	FeSO_4_ + H_2_O_2_, 40–50 °C	- More efficient digestion of organic matter than H_2_O_2_ alone. - Mild reaction conditions minimize polymer damage.	- May cause minor degradation in biodegradable polymers (e.g., PLA). - An orange precipitates require removal (citric acid or density separation).	[33,72,75,80,81,82]
Alkaline digestion	NaOH (1–10 M), KOH (10–20%), 20–80 °C	- Efficient at decomposing proteins, lipids, and carbohydrates into a low-molecular-weight aqueous solution.	- Degradation of PC, and PET under concentrated or prolonged exposure. - The structure of PC and PET makes them susceptible to saponification and mass loss.	[70,72,75,83]
Acid digestion	15.7 M HNO_3_, HClO_4_, H_2_SO_5_, 37% HCl	- Effective at destroying biogenic matter. - High efficiency in removing biological matter.	- Destructive to several polymers (PA, PUR, ABS, PMMA and PVC). - Alters surface morphology, degraded PA, altered the surface of PVC, and caused PET to agglomerate	[72,84,85]
Enzymatic digestion	Protease K, cellulase, chitinase	- Highly effective at removing biogenic matter. - Minimal polymer degradation.	- Long incubation periods (days to weeks). - Fenton’s reagent be incorporated at an intermediate stage of the process	[72,80]
Density separation	ZnCl_2_ (ρ = 1.6–1.8 g/cm^3^), NaCl, NaBr, NaI, KI	- Efficient separation based on polymer density. - Compatible with most oxidation and digestion methods. - ZnCl_2_ achieves high recovery rates.	- ZnCl_2_ has a negative environmental impact. - NaCl and NaBr have lower density; limited separation for dense polymers. - NaI and KI are costly.	[31,72,86,87,88]
Oleoextraction	Sunflower oil + H_2_O_2_; hexane + ethanol washing	- Less aggressive technique. - Broad density range (including polymers ≈ 2 g/cm^3^). - Prevents salt residues that interfere with polymer identification in subsequent analysis stages.	- Requires multiple solvent steps. - New technique; still limited validation across sample types.	[73,89]

**Table 3:** Microplastic concentrations and characteristics of the analyzed studies.

City	Microplastic Concentration	Types of MP	MP Sizes Range (µm)	Ref.
Lhasa, China	0.15–0.25 MP/m^3^	PE, PP	10–100	[21]
Beijing, China	123.6 MP/g	PP, PA, PE, PET, PC, Silicone	20–100	[65]
New Jersey, USA	Fibers: 120–158 MP/m^2^ Films: 102–126 Fragments: 62–86	PS, PET, PVC, PE, PP	35–100	[25]
Asaluyeh, Iran	Passive: 60 MP/g		100–250	[64]
London, England	575–1008 MP/m^2^	PP, PVC, PE, PET, PS, PUR, PAN, PES, PA, Acrylic	400–500	[23]
Seoul, South Korea	103 MP/m^3^	PET, PP, PE, PEVA, PS, PU	<100	[33]
Hamburg, Alemania	-	PE, EVAC, PTFE, PVA, PET	63–5000	[24]
Sakarya, Turkey	Depending on the sampling day, the color and shape of microplastics	PA, PL	-	[66]
Coastal city, Eat China	Indoor: 1583 MP/m^3^ Outdoor: 189 MP/m^3^	PE, PS, PL	<100	[15]
Wageningen, Amerongen, Zetten and Utrecht. Netherlands	96,000 MP/m^3^	PET y/o PU, PA, PP, PVC, POM, PMMA	-	[67]
Spain and the Canary Islands	22.3–29.6 MP/m^2^	PS, acrylic polymers, PP, alkyd resins, PES, PA, PVC, PSU	67.7–72.4	[22]
Netherlands	1.2–305 mg MP/g	PET	-	[68]
Shanghai, China	PET: 1550–120,000 mg/kg PC: 4.6 mg/kg	PET, PC	50–2000	[69]
Shanghai, China	15–94 MP/m^3^	PE, PES, PVC, PP, PU, rubber	240–2181.48	[70]

## Data Availability

No new data were created or analyzed in this study. Data sharing is not applicable to this article.
